# Synthesis of Xylooligosaccharides of Daidzein and Their Anti-Oxidant and Anti-Allergic Activities

**DOI:** 10.3390/ijms12095616

**Published:** 2011-08-31

**Authors:** Kei Shimoda, Hiroki Hamada, Hatsuyuki Hamada

**Affiliations:** 1 Department of Chemistry, Faculty of Medicine, Oita University, 1-1 Hasama-machi, Oita 879-5593, Japan; 2 Department of Life Science, Faculty of Science, Okayama University of Science, 1-1 Ridai-cho, Kita-ku, Okayama 700-0005, Japan; 3 National Institute of Fitness and Sports in Kanoya, 1 Shiromizu-cho, Kagoshima 891-2390, Japan; E-Mail: hamajudo2@hotmail.com

**Keywords:** daidzein, xylooligosaccharide, biocatalyst, anti-oxidant activity, anti-allergic activity

## Abstract

The biocatalytic synthesis of xylooligosaccharides of daidzein was investigated using cultured cells of *Catharanthus roseus* and *Aspergillus* sp. β-xylosidase. The cultured cells of *C. roseus* converted daidzein into its 4′-*O*-β-glucoside, 7-*O*-β-glucoside, and 7-*O*-β-primeveroside, which was a new compound. The 7-*O*-β-primeveroside of daidzein was further xylosylated by *Aspergillus* sp. β-xylosidase to daidzein trisaccharide, *i.e.*, 7-*O*-[6-*O*-(4-*O*-(β-d-xylopyranosyl))-β-d-xylopyranosyl]-β-d-glucopyranoside, which was a new compound. The 4′-*O*-β-glucoside, 7-*O*-β-glucoside, and 7-*O*-β-primeveroside of daidzein exerted DPPH free-radical scavenging and superoxide radical scavenging activity. On the other hand, 7-*O*-β-glucoside and 7-*O*-β-primeveroside of daidzein showed inhibitory effects on IgE antibody production.

## 1. Introduction

Daidzein is one of the most important soy isoflavonoids and has been recognized as a natural antioxidant. It has been reported to show anti-allergic activities such as inhibitory effects on histamine release from mast cells [1–5]. However, their use as food-ingredients is limited because of their water-insolubility and low absorbability after oral administration.

Biocatalytic glycosylation using cultured cells and enzymes as biocatalysts is useful for preparing water-soluble and stable glycosides from water-insoluble and unstable organic compounds [6–11]. Furthermore, it has been reported that glycosylation of a lipophilic flavonoid, *i.e.*, quercetin, improved its absorbability after oral administration [12]. From the viewpoint of pharmacological development of isoflavonoids, their glycosylation is of importance. We report here the synthesis of glycosides of daidzein such as 7-*O*-β-primeveroside and 7-*O*-[6-*O*-(4-*O*-(β-d-xylopyranosyl))-β-d-xylopyranosyl]- β-d-glucopyranoside, which were two new compounds, by biocatalytic glycosylation of daidzein with cultured cells of *Catharanthus roseus* and *Aspergillus* sp. β-xylosidase. We also report their anti-oxidant activities such as 2,2-diphenyl-1-picrylhydrazyl (DPPH) radical scavenging activity and superoxide-radical scavenging activity, and the inhibitory activity for IgE antibody formation.

## 2. Results and Discussion

### 2.1. Biocatalytic Glycosylation of Daidzein (**1**) by Cultured Cells of *C. roseus* and *Aspergillus* sp. β-Xylosidase

HPLC analyses of the MeOH extracts of cultured cells of *C. roseus* revealed that neither daidzein nor its glycosides existed in the cell cultures. After incubation of cultured cells of *C. roseus* with daidzein (**1**) for five days, the products **2**–**4** were isolated from the cells by extraction with MeOH. No additional products were detected in the MeOH extracts of the cells despite careful HPLC analyses.

**Figure 1:**
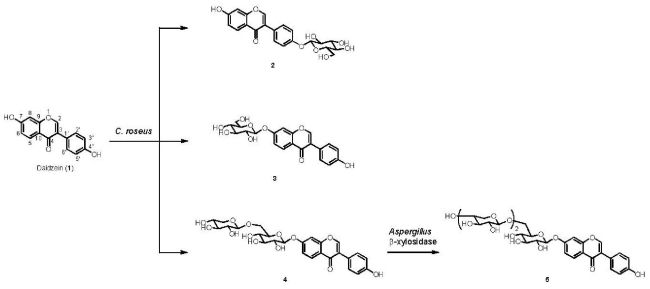
Synthesis of β-xylooligosaccharides of daidzein (**1**) by *C. roseus* and *Aspergillus* β-xylosidase.

On the basis of their HRFABMS, ^1^H and ^13^C NMR (Table 1), H-H COSY, C-H COSY, and NOE-spectroscopic analyses, the products were determined to be daidzein 4′-*O*-β-glucoside (**2**, 2%), daidzein 7-*O*-β-glucoside (**3**, 30%), and daidzein 7-*O*-β-primeveroside (**4**, 5%) (Figure 1), of which **4** is new.

The molecular formula of **4** was established as C_26_H_28_O_13_ based on its HRFABMS spectrum, which included a pseudomolecular ion [M + Na]^+^ peak at *m*/*z* 571.1205. HRFABMS suggested that **4** was composed of one of each of the following molecules of **1**: hexose, and pentose. Its ^1^H NMR spectrum showed two anomeric proton signals at δ 4.20 (1H, *d*, *J* = 8.0 Hz) and 5.10 (1H, *d*, *J* = 7.6 Hz). This suggested the presence of two β-anomers. The ^13^C NMR spectrum included two anomeric carbon signals at δ 99.9 and 103.5. The sugar components of **4** were determined to be β-d-glucopyranose and β-d-xylopyranose based on the chemical shifts of the carbon signals. The ^13^C resonance of C-6″ was shifted downfield to δ 68.7. Correlations were observed between the anomeric proton signal at δ 5.10 (H-1″) and the carbon signal at δ 161.3 (C-7), and between the anomeric proton signal at δ 4.20 (H-1‴) and the carbon signal at δ 68.7 (C-6″) in the HMBC spectrum. These findings confirmed that the inner glucopyranosyl residue was attached to the phenolic hydroxyl group at C-7 of daidzein (**1**), and that the pair of β-d-glucopyranosyl residue and β-d-xylopyranosyl residue was 1,6-linked. Thus, **4** was identified as daidzein 7-*O*-[6-*O*-(β-d-xylopyranosyl)]-β-d-glucopyranoside (7-*O*-β-primeveroside).

Enzymatic glycosylation of daidzein 7-*O*-β-primeveroside (**4**) using β-xylosidase from *Aspergillus* sp. as a biocatalyst afforded product **5** (Figure 1). The structure of product **5** was identified on the basis of HRFABMS, ^1^H and ^13^C NMR (Table 1), H-H COSY, C-H COSY, and HMBC-spectra as daidzein 7-*O*-[6-*O*-(4-*O*-(β-d-xylopyranosyl))-β-d-xylopyranosyl]-β-d-glucopyranoside (15%), which is a new compound.

Product **5** was assigned an *Mr* of 703.1902 [M + Na]^+^ in the HRFABMS spectrum, which suggested a molecular formula of C_31_H_36_O_17_. In the ^13^C NMR spectrum of **5**, the chemical shifts of the sugar carbon signals indicated that the sugar components in **5** were β-d-glucopyranose and β-d-xylopyranose. Correlations were observed in the HMBC spectrum between the proton signal at δ 5.11 (H-1″) and the carbon signal at δ 161.3 (C-7), between the proton signal at δ 4.51 (H-1‴) and the carbon signal at δ 68.8 (C-6″), and between the proton signal at δ 4.22 (H-1‴′) and the carbon signal at δ 78.2 (C-4‴). These results confirmed that the inner β-d-glucopyranosyl residue was attached to the phenolic hydroxyl group at C-7 of daidzein, that second β-d-xylopyranosyl residue and inner β-d-glucopyranosyl residue were 1,6-linked, and that the third and second β-d-xylopyranosyl residues were 1,4-linked. Thus, compound **5** was identified as daidzein 7-*O*-[6-*O*-(4-*O*-(β-d-xylopyranosyl))-β-d-xylopyranosyl]-β-d-glucopyranoside.

### 2.2. Anti-Oxidant Activity of Glycosides of Daidzein

The DPPH free-radical scavenging activity of daidzein (**1**) and its β-glycosides **2**–**5** was examined by *in vitro* bioassay. The antioxidant activities were expressed as IC_50_ values summarized in Table 2. Daidzein 4′-*O*-β-d-glucoside (**2**) and daidzein 7-*O*-β-d-glucoside (**3**) showed strong DPPH free-radical scavenging activity. As compared with the antioxidative property of daidzein, glucosylation of daidzein slightly decreased its antioxidant activity. Daidzein 7-*O*-β-primeveroside (**4**) had relatively low antioxidant activity. The results obtained here suggested that glucosides and primeveroside of daidzein would be potent free-radical scavenging antioxidants with aqueous-solubility.

The superoxide-radical scavenging activity of daidzein β-glycosides **2**–**5** was expressed as IC_50_ values and were summarized in Table 2. The activity of daidzein 4′-*O*-β-d-glucoside (**2**) and daidzein 7-*O*-β-d-glucoside (**3**) was strong and that of daidzein 7-*O*-β-primeveroside (**4**) was low. Daidzein 7-*O*-[6-*O*-(4-*O*-(β-d-xylopyranosyl))-β-d-xylopyranosyl]-β-d-glucopyranoside (**5**) rarely showed superoxide-radical scavenging activity. The results obtained here suggested that glucosides of daidzein would be potential superoxide-radical scavenging antioxidants. Studies on Trolox equivalent antioxidant capacity (TEAC) of the glycosides are now in progress.

### 2.3. Anti-Allergic Activity of Glycosides of Daidzein

The effects of daidzein (**1**) and its β-glycosides **2**–**5** on IgE antibody formation were examined by *in vivo* bioassay using 7S-globulin from soybean as an antigen. The average rat plasma IgE level after treatment of 7S-globulin with or without test compounds is summarized in Table 3. Daidzein showed the highest anti-allergic activity among the compounds tested. Daidzein 7-*O*-β-d-glucoside (**3**) and daidzein 7-*O*-β-primeveroside (**4**) showed inhibitory action on IgE antibody generation. On the other hand, daidzein 4′-*O*-β-glucoside (**2**) and daidzein 7-*O*-[6-*O*-(4-*O*-(β-d-xylopyranosyl))-β-d-xylopyranosyl]- β-d-glucopyranoside (**5**) did not inhibit the IgE antibody formation.

Recently, it has been reported that 7-*O*-β-glycosides of genistein showed anti-allergic activity, *i.e.*, inhibitory action on histamine release from rat peritoneal mast cells, whereas the β-glycosides, the sugar of which attached at other phenolic hydroxyl groups, exhibited no anti-allergic actions [13]. The results of the present study suggested that β-glucoside and β-primeveroside at C-7 of daidzein did not attenuate the anti-allergic activity, and that phenolic hydroxyl groups at 4′-position might be necessary for the anti-allergic action of glycosides of daidzein. Studies on the mechanism of β-glycosides of daidzein to act as anti-allergic formulations are now in progress.

### 2.4. Water-Solubility of Glycosides of Daidzein

The water-solubility of daidzein (**1**) and its β-glycosides **2**–**5** was examined and summarized in Table 4. In the case of daidzein 7-*O*-[6-*O*-(4-*O*-(β-d-xylopyranosyl))-β-d-xylopyranosyl]-β-d-glucopyranoside (**5**), conjugation of daidzein to three glycoside units enhanced the water-solubility to 1.0 μmol/mL, which was 850 fold higher than that of **1** (1.2 × 10^−3^ μmol/mL).

## 3. Experimental Section

### 3.1. General

Daidzein was purchased from Sigma-Aldrich Co. *Aspergillus* sp. β-xylosidase was obtained from Dr. Otsuka of Okayama University of Science. The NMR spectra were recorded in DMSO-*d*_6_ using a Varian XL-400 spectrometer. The chemical shifts were expressed in δ (ppm) referring to tetramethylsilane. The HRFABMS spectra were measured using a JEOL MStation JMS-700 spectrometer. HPLC was carried out on a YMC-Pack R&D ODS column (150 × 30 mm) [solvent: CH_3_CN:H_2_O (3:17, v/v); detection: UV (280 nm); flow rate: 1.0 mL/min].

### 3.2. Cell Line and Culture Conditions

The cultured plant cells of *C. roseus* have been cultivated over 20 years in our laboratory and subcultured in 300 mL conical flasks containing Schenk and Hildebrand (SH) medium (100 mL, pH 5.7) on a rotary shaker (120 rpm) at 25 °C in the dark for every 3–5 weeks. Part of the callus tissues (fresh weight 30 g) was transplanted to freshly prepared SH medium (100 mL in a 500 mL conical flask, pH 5.7) containing 3% sucrose and was incubated for 3 weeks prior to use for this work.

### 3.3. Glycosylation of Daidzein by *C. roseus*

Daidzein (0.08 mmol) dissolved in EtOH 300 μL was individually administered to a 500-mL flask containing suspension cultured cells of *C. roseus*. The cultures were then incubated at 25 °C for five days on a rotary shaker (120 rpm) under illumination. After incubation, the cells and medium were separated by filtration with suction. The filtered medium was extracted with EtOAc. The medium was further extracted with *n*-BuOH. EtOAc and *n*-BuOH fractions were analyzed by HPLC The cells were extracted with MeOH for 12 h and sonicated for 5 min. The yields of the glycosylation products were calculated on the basis of the peak area from HPLC using calibration curves prepared by HPLC analyses of the authentic glycosides. The MeOH fraction was conc. and partitioned between H_2_O and EtOAc. The EtOAc fractions were combined and analyzed by the HPLC. The H_2_O fraction was applied to a Diaion HP-20 column and the column was washed with H_2_O followed by elution with MeOH. The MeOH eluate was subjected to HPLC to give glycosylated products.

Spectral data of a new compound, daidzein 7-*O*-β-primeveroside (**4**): HRFABMS: *m*/*z* 571.1205 [M + Na]^+; 1^H NMR (DMSO-*d*_6_): δ 3.15–3.70 (11H, *m*, H-2″, 2‴, 3″, 3‴, 4″, 4‴, 5″, 5‴, 6″), 4.20 (1H, *d*, *J* = 8.0 Hz, H-1‴), 5.10 (1H, *d*, *J* = 7.6 Hz, H-1″), 6.82 (2H, *d*, *J* = 6.4 Hz, H-3′, 5′), 7.12 (1H, *dd*, *J* = 8.6, 2.0 Hz, H-6), 7.24 (1H, *d*, *J* = 1.9 Hz, H-8), 7.40 (2H, *d*, *J* = 6.4 Hz, H-2′, 6′), 8.05 (1H, *d*, *J* = 8.6 Hz, H-5), 8.39 (1H, *s*, H-2); ^13^C NMR (DMSO-*d*_6_): see Table 1.

### 3.4. Glycosylation of Daidzein 7-*O*-β-Primeveroside by *Aspergillus* sp. β-Xylosidase

The transglycosylation reaction using *Aspergillus* sp. β-xylosidase was carried out at 37 °C in 25 mM sodium phosphate buffer. To a solution containing 0.1 mmol of daidzein 7-*O*-β-primeveroside and 5 mmol of xylobiose in 25 mM of HEPES-NaOH buffer (pH 7.5) was added 100 U of β-xylosidase. After stirring of the reaction mixture for 24 h, the mixture was centrifuged at 3000 g for 10 min. The supernatant was subjected on to a Sephadex G-25 column equilibrated with water to remove the enzyme. The fractions containing glycosides were purified by preparative HPLC to give a product.

Spectral data of a new compound, daidzein 7-*O*-[6-*O*-(4-*O*-(β-d-xylopyranosyl))-β-d-xylopyranosyl]- β-d-glucopyranoside (**5**): HRFABMS: *m*/*z* 703.1902 [M + Na]^+; 1^H NMR (DMSO-*d*_6_): δ 3.09–3.75 (16H, *m*, H-2″, 2‴, 2‴′, 3″, 3‴, 3‴′, 4″, 4‴, 4‴′, 5″, 5‴, 5‴′, 6″), 4.22 (1H, *d*, *J* = 8.0 Hz, H-1‴′), 4.51 (1H, *d*, *J* = 8.0 Hz, H-1‴), 5.11 (1H, *d*, *J* = 7.6 Hz, H-1″), 6.82 (2H, *d*, *J* = 6.4 Hz, H-3′, 5′), 7.13 (1H, *dd*, *J* = 8.6, 2.0 Hz, H-6), 7.24 (1H, *d*, *J* = 2.0 Hz, H-8), 7.41 (2H, *d*, *J* = 6.4 Hz, H-2′, 6′), 8.04 (1H, *d*, *J* = 8.6 Hz, H-5), 8.39 (1H, *s*, H-2); ^13^C NMR (DMSO-*d*_6_): see Table 1.

### 3.5. DPPH Radical Scavenging Activity

DPPH free-radical scavenging activities of β-glycosides of daidzein were determined as follows. DPPH was dissolved in ethanol (500 μM) [14]. The sample solutions were prepared by dissolving each compound in ethanol. To a solution containing 0.1 mL of various concentration of each sample and 0.9 mL of ethanol was added 1 mL of DPPH solution at room temperature. Vitamin C was used as a positive control. After 20 min at 25 °C, the absorbance was measured at 517 nm. The percentage reduction of the initial DPPH adsorption, *i.e.*, the free-radical scavenging activity, was calculated as follows: *E* = [(*A*_c_ − *A*_t_)/*A*_c_] × 100, where *A*_t_ and *A*_c_ are the respective absorbance at 517 nm of sample solutions with and without the test compounds. Antioxidant activity was expressed as the 50% inhibitory concentration (IC_50_).

### 3.6. Superoxide-Radical Scavenging Activity

Superoxide was generated by the xanthine-xanthine oxidase system [14]. The reaction mixture contained 50 μL of 4 mM xanthine, 50 μL of various concentration of sample in ethanol, 50 μL of 2 mM nitro blue tetrazolium (NBT), 50 μL of 0.3 nkat/mL xanthine oxidase, and 0.1 M phosphate buffer (pH 7.4) in a total volume of 2 mL. Vitamin C was used as a positive control. The reaction mixture was incubated at 25 °C for 10 min and the absorbance was read at 560 nm. Percent inhibition was calculated by comparing with control without test compound but containing the same amount of alcohol. The IC_50_ value is shown as the sample concentration at which 50% of superoxide-radical was scavenged.

### 3.7. Suppressive Action on IgE Antibody Formation

The inhibitory action of β-glycosides of daidzein on IgE antibody formation was examined as follows. 7S-Globulin was used as the antigen (1 mg/rat), and Al(OH)_3_ and pertussis vaccine were used as the adjuvants (20 mg and 0.6 mL/rat, respectively). Sensitization was made by injection of a mixture (0.6 mL) of the antigen and the adjuvant into the paws of each rat (male, ca. 200 g). Paw edema was measured 24 h after injection and the treated rats were divided in groups with an equal average swelling volume. Each sample was dissolved in physiological saline containing 10% Nikkol and the solution was injected daily into the rat for 11 d starting on the day of grouping. Hydrocortisone was used as the positive control. The amount of IgE was measured by the passive cutaneous anaphylaxis method on the 15th day [15]. The results were expressed as average of plasma IgE level of 7 rats administered a total of 10 mg/kg of each test compound.

### 3.8. Water-Solubility

Water-solubility of daidzein and its β-glycosides was examined as follows. Each compound was stirred in water for 24 h at 25 °C. The mixture was centrifuged at 100,000 g for 30 min at 25 °C. The concentration of test compounds was estimated on the basis of their peak areas using calibration curves prepared by HPLC analyses of authentic samples.

## 4. Conclusions

The xylooligosaccharides of daidzein, that is, water-soluble daidzein derivatives, were synthesized by sequential biocatalytic glycosylation with cultured cells of *C. roseus* and β-xylosidase from *Aspergillus* sp. The β-glucosides and β-primeveroside of daidzein exerted DPPH free-radical scavenging and superoxide radical scavenging activity. The 7-*O*-β-glucoside and 7-*O*-β-primeveroside of daidzein showed inhibitory effects on IgE antibody production. Further studies on the basic toxicological properties such as anticancer activity of the newly synthetic compounds are now in progress.

## Figures and Tables

**Table 1:** ^13^C chemical shifts of the glycosylation products **2**–**5** in DMSO-*d*_6_.

Product	2	3	4	5
2	153.0	153.1	153.1	153.1
3	122.0	122.0	122.1	122.1
4	174.6	174.6	174.6	174.6
5	127.0	127.0	127.0	127.0
6	115.5	115.5	115.4	115.4
7	161.2	161.3	161.3	161.3
8	103.3	103.3	103.3	103.3
9	157.2	157.2	157.2	157.1
10	118.4	118.4	118.4	118.4
1′	123.6	123.6	123.6	123.6
2′	130.0	130.0	130.0	130.0
3′	114.9	114.9	114.9	114.9
4′	157.0	156.9	156.9	156.9
5′	114.9	114.9	114.9	114.9
6′	130.0	130.0	130.0	130.0
1″	99.5	99.9	99.9	99.5
2″	73.0	73.0	73.3	73.0
3″	77.0	77.0	76.0	76.8
4″	69.5	69.8	69.9	69.5
5″	76.4	76.1	75.9	76.4
6″	60.7	60.5	68.7	68.8
1‴			103.5	103.5
2‴			72.9	72.8
3‴			76.5	76.1
4‴			69.3	78.2
5‴			60.9	61.0
1‴′				103.6
2‴′				72.9
3‴′				76.7
4‴′				68.9
5‴′				60.8

**Table 2:** Antioxidant activities of daidzein β-glycosides **2**–**5**.

Compound		IC_50_ (μM)

	DPPH Free-Radical Scavenging	Superoxide-Radical Scavenging
**1**	50	751
**2**	70	802
**3**	55	767
**4**	121	870
**5**	177	908
Vitamin C	38	698

**Table 3:** Suppressive action of daidzein β-glycosides **2**–**5** on IgE antibody formation.

Compound	IgE Level a
None	410.0
**1**	141.7
**2**	422.5
**3**	158.0
**4**	197.7
**5**	339.8
Hydrocortisone	338.0

a The results were expressed as average of plasma IgE level of 7 rats administered a total of 10 mg/kg of each test compound.

**Table 4:** Water-solubility of daidzein (**1**) and its β-glycosides **2**–**5**.

Compound	Water-Solubility (μmol/mL)	Fold a
**1**	1.2 × 10^−3^	1
**2**	3.5 × 10^−2^	29
**3**	4.0 × 10^−2^	33
**4**	0.2	183
**5**	1.0	850

a Fold of water-solubility of glycosides **2**–**5** is expressed relative to that of their aglycone **1**, normalized to 1.
